# Burkitt's Lymphoma in a Pregnant Woman: Case Report and Review of the Literature

**DOI:** 10.1155/2013/370179

**Published:** 2013-05-12

**Authors:** Carlos Zagalo, Francesca Pierdomenico, José Cabeçadas, Pedro David Santos

**Affiliations:** ^1^Egas Moniz, Health Sciences Institute, Quinta da Granja, Monte de Caparica, 2829-511 Caparica, Portugal; ^2^Serviço de Hematologia, Instituto Português de Oncologia de Lisboa Francisco Gentil EPE, Rua Professor Lima Basto, 1099-023 Lisboa, Portugal; ^3^Serviço de Anatomia Patológica, Instituto Português de Oncologia de Lisboa Francisco Gentil EPE, Rua Professor Lima Basto, 1099-023 Lisboa, Portugal; ^4^Instituto Ciências Biomédicas Abel Salazar, Universidade do Porto, Rua de Jorge Viterbo Ferreira No. 228, 4050-313 Porto, Portugal

## Abstract

Burkitt's lymphoma (BL) is an aggressive B-cell malignancy with very high proliferation rate, more common in males than females. Here, we describe a case of Burkitt's lymphoma in a 24-week pregnant woman with cervical and abdominal involvement. The common genetic event of virtually all BL is a reciprocal chromosomal translocation involving the proto-oncogene *MYC* and one of the Ig gene heavy or light chain loci. Supportive treatment was administered until early delivery, after which the patient was treated according to protocol LMB96. Pregnancy and tumorogenesis share some important events such as immunologic tolerance, angiogenesis, and editing the host immune response. Little is known about the relationship between these events in pregnancy and in tumorogenesis.

## 1. Introduction

Burkitt's lymphoma was first described in 1957 by the Irish surgeon Dennis Parsons Burkitt, after observation of a so-called “sarcoma of the jaws” in children in Uganda, Africa. Burkitt's lymphoma (BL) is a highly aggressive non-Hodgkin lymphoma (NHL), with a very fast growth rate, often affecting extranodal sites or with leukemic presentation [1]. Burkitt's lymphoma is usually diagnosed in children and young adults and, to a lesser extent, in middle-aged adults [2]. Based on epidemiology, three different forms of Burkitt's lymphoma have been described until now, an endemic form, a sporadic form, and one related to HIV infection. The endemic form (very common in Africa and Papua New Guinea) is strongly associated (95%) with Epstein-Barr virus (EBV) infection and malaria [3] and usually affects children that present with facial bones involvement.

The sporadic form (5–15% associated with EBV) affects mainly abdominal viscera, terminal ileum, caecum, mesentery, and Waldeyer ring; the third variant is usually associated with HIV infection, and 45% of patients are coinfected with EBV. Burkitt's lymphoma is more common in males than females [4]. 

The common genetic event of virtually all BL is a reciprocal chromosomal translocation involving the proto-oncogene *MYC *(on chromosome 8) and one of the Ig gene heavy or light chain loci (on chromosomes 2, 14, or 22) [4]. The vast majority of Burkitt's lymphomas exhibit t(8; 14)(q24; q32). As a result of the reciprocal chromosomal translocation, the oncogene *c-myc* is overexpressed under the influence of Ig enhancers regulatory regions. Constitutive expression of *c-myc* oncogene promotes an increased B-cell proliferation, as well as a reduced capacity for induction of apoptosis [5].

Burkitt's lymphoma/leukemia was one of the first malignancies shown to be curable with intensive chemotherapy, and the majority of children and young adult patients are curable today with aggressive combination chemotherapy regimens [2]. 

## 2. Case Presentation

A twenty-five years old woman, in the 24th week of gestation, presented with a voluminous cervical mass located on the left front neck. She had first noticed an enlargement of her neck 3 months earlier, and, apart from tiredness, she was completely asymptomatic. 

On observation, she had an isolated cervical mass of about 5 × 10 cm long, hard on palpation, not mobile.

**Figure 1:**
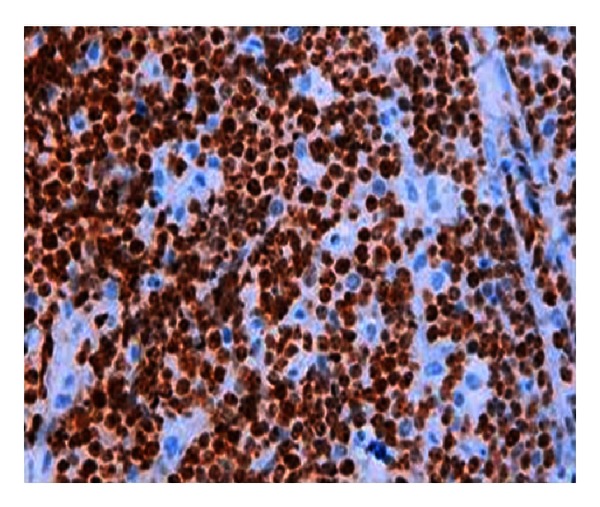
Ki67 almost 100%.

Fine needle aspiration showed a high grade B-cell lymphoma, CD10+ (flow cytometry), and the biopsy to the cervical lesion confirmed the diagnosis of Burkitt's lymphoma with gene break point on MYC (8q24) determined by FISH (fluorescent in situ hybridization). The tumour was CD20 (+), CD10 (+), Ki67 (+) (>90% of cells) (Figure 1), CD3 (−), BCL2 (−), and TdT (−). The patient is HIV-1 negative, HIV-2 negative, EBV Ig G positive, and EBV Ig M negative.

**Figure 2:**
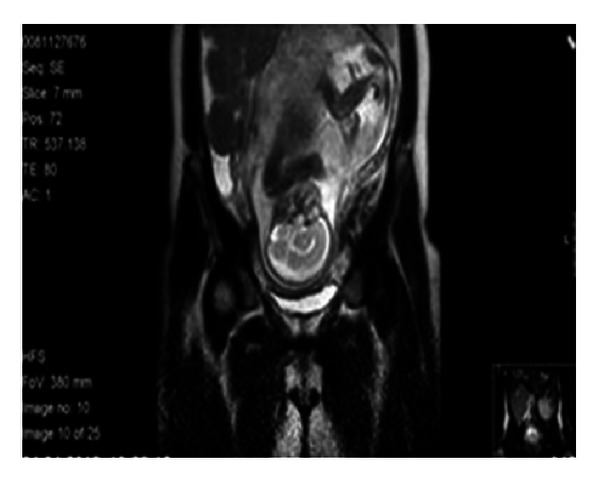
MR coronal cut revealing a fetus and the abdominal tumour mass.

As she was pregnant at the time of diagnosis, a total body MRI (magnetic resonance imaging) was used for staging instead of conventional CT scan. MRI revealed a cervical mass of 104 × 63 × 75 mm deviating the trachea and oesophagus leftwards and in the infra diaphragmatic compartment, a mass measuring 135 × 116 × 94 mm in the right flank and a fetus of approximately 25 weeks (Figure 2). There were no bone marrow infiltration and no central nervous system (CNS) infiltration, and the LDH level was elevated (1376 *μ*L/L). According to the Murphy/St. Jude staging classification for Burkitt's lymphoma, the patient was in stage III. In an intention to safely carry on the pregnancy until week 28 of gestation, under monitoring by an obstetrician, chemotherapy with agents potentially less harmful for the baby was proposed. Cyclophosphamide, doxorubicin, vincristine, prednisone, plus rituximab (R-CHOP) was performed with reduction of the cervical mass; however, in two weeks' time, the mass started growing again and LDH increased. Delivery was performed at 28th week of gestation +5, and a healthy baby was born by caesarean section. After delivery, the patient proceeded with conventional treatment for BL according to LMB-96 protocol, group B [6], without major complications.

Assessment of treatment response after R-CYM (cytarabine, methotrexate, and rituximab) by CT and PET scan showed complete remission, and the patient completed chemotherapy according to LMB96 group B protocol in August 2012, with persisting complete remission. 

## 3. Discussion

Whereas *MYC* translocation is found in almost all BL, it may be seen as well in some cases of diffuse large B-cell lymphoma (DLBCL) which is much more common, and may have a different treatment approach [7]. 

Burkitt's lymphoma's brief and intense treatment regimens have led to high cure rates even in adults, especially when immunotherapeutic approach is used [8]. Rituximab is a monoclonal antibody specific to CD20 that induces death on B cells by antibody-dependent cell-mediated cytotoxicity [9]. Early diagnosis and intense and short treatment regimens are ways of increasing cure rates [8].

This is an interesting case not only because it was not possible to determine if it is purely extranodal BL (most common), but also because the patient was pregnant.

A diagnosis of Burkitt's lymphoma during pregnancy implies difficulties both in staging procedures and in treatment decision. Conventional staging methods as CT and PET scan should not be performed, and BL treatment protocol such as LMB-96 includes high-dose methotrexate and cytarabine which are contraindicated during pregnancy. Using alternative diagnostic means, chemotherapy regimens with drugs potentially less harmful for the baby and a close followup together with obstetric consult are crucial. 

Immunologic tolerance, establishment of a nutrient supply system (angiogenesis), and evasion or editing the host immune response are essential steps for a normal pregnancy and also for tumorogenesis. Epithelial-mesenchymal transition, loss of integrin, and E-cadherin expression are essential for the invasion processes in pregnancy and tumor growth. Vasculogenic mimicry (nonendothelial cell assumes form and function of a vascular structure) is a process observed in extra villous trophoblast (EVT) cells (invasion of spiral arteries of maternal decidua, early in placental development) and in some aggressive melanoma cells, sharing the same event—expression of galectin 3 [10]. Immunologic modulation is also a phenomenon encountered both in pregnancy, and in tumor proliferation which allows the presence, growth, and development of semiallogeneic cells (fetus and tumour). 40% of cells of the decidua, in a pregnant uterus, are immune cells (NK, macrophages and dendrocytes). Uterus NK (70% of uterus immune cells) are CD16 (−) which means that they lack cytotoxic capacity. They also secrete galectin-1, responsible for induction of a tolerant behaviour in dendritic cells (DC). CD16 (−) NK cells infiltrated in tumours, like renal cell carcinoma, have been taken as responsible for reducing cytotoxic activity and modulating DC immune activity on these tumours [10]. Regulatory T cells (present both in normal pregnancy and cancer) have an important role in increasing tumor blood vessel density and in impaired antitumor immunity [10]. Still very little is known about the relationship between these events in pregnancy and in tumorogenesis.
